# The Maize Calcium-Binding Protein ZmCML36 Positively Regulates Tolerance to Drought and Salt Stress

**DOI:** 10.3390/plants15213409

**Published:** 2026-11-02

**Authors:** Zixin Zhang, Pengxiang Bao, Xinyue Zhang, Tingting Gao, Yuhang Zhang, Zhiqiang Wu, Meiyi Liu, Zhenyuan Zang, Heng Pan, Liangyu Jiang, Renhai Jiao, Xin Qi

**Affiliations:** 1 College of Agriculture, Jilin Agricultural University, Changchun 130118, China; 2 Jilin Academy of Agricultural Sciences (Northeast Agricultural Research Center of China), Changchun 130033, China

**Keywords:** maize, *ZmCML36*, calmodulin-like protein, drought stress, salt stress

## Abstract

Drought and salinity severely limit maize (*Zea mays* L.) production worldwide. Calcium (Ca^2+^) signaling is central to plant stress responses, but the functions of many calcium sensors, including calmodulin-like proteins (CMLs), remain poorly understood in maize. Here, we characterized ZmCML36 as a positive regulator of drought and salt stress tolerance. Transcriptional analysis showed that *ZmCML36* was strongly induced by drought and salt treatments. Overexpression of *ZmCML36* in *Arabidopsis* and maize enhanced stress tolerance, as indicated by higher germination rates, longer roots, increased superoxide dismutase (SOD) activity, and reduced malondialdehyde (MDA) content. Conversely, an EMS-derived *ZmCML36* mutant exhibited greater stress sensitivity. Transient expression analysis showed ZmCML36-GFP fluorescence at the cell periphery and nuclear region. An in vitro mobility-shift assay supported the Ca^2+^-binding capacity of ZmCML36. A yeast two-hybrid screen, followed by luciferase complementation imaging in *Nicotiana benthamiana*, identified an interaction between ZmCML36 and Zm00001eb395720, a putative regulatory β subunit of the maize SnRK1 complex. These findings suggest a potential connection between Ca^2+^ signaling and SnRK1-associated energy regulation during abiotic stress responses and identify *ZmCML36* as a candidate gene for improving abiotic stress tolerance in maize.

## 1. Introduction

Drought and salinity are major abiotic constraints on maize production because they disrupt plant growth, development, and productivity [1,2,3,4,5,6]. Drought-related crop losses can exceed those caused by all plant pathogens combined [7]. Understanding the molecular mechanisms that enable maize to respond to these stresses is therefore important for identifying stress-tolerance genes and developing crops that are more resilient to environmental change [8,9,10,11,12].

Plants respond to environmental stress through complex signal-transduction networks. Cytosolic calcium (Ca^2+^) is a central second messenger in these networks, and a transient increase in cytosolic Ca^2+^ is among the earliest events following stress perception [13,14,15]. Calcium-binding proteins decode these signals, including calmodulins (CaMs), calcium-dependent protein kinases (CDPKs), calcineurin B-like proteins (CBLs), and calmodulin-like proteins (CMLs) [16,17]. Canonical CaM is highly conserved and contains four EF-hand motifs arranged in two pairs. CMLs are more diverse in sequence and in the number and organization of EF-hand motifs, providing a basis for differences in Ca^2+^ sensing and target recognition [14,18,19,20].

CML proteins have diverse functions in plant growth, development, and abiotic stress responses [14,16,21]. Recent functional analysis of *ZmCML3* also supports a role for maize CML proteins in drought tolerance [22]. In alfalfa (*Medicago sativa*), overexpression of *MsCML70* in *Arabidopsis* enhanced salt tolerance by affecting several biological pathways and downstream stress-responsive genes [23]. Some CMLs contribute to salt tolerance by regulating ion homeostasis or the accumulation of compatible solutes such as proline [24,25]. In rice (*Oryza sativa*), *OsDSR-1* positively regulates drought tolerance by enhancing reactive oxygen species (ROS) scavenging and increasing sensitivity to abscisic acid [26]. In wild tomato (*Solanum habrochaites*), overexpression of *ShCML44* enhanced tolerance to drought, salinity, and cold by increasing antioxidant enzyme activity and improving plant water relations [27].

Despite progress in characterizing the CML family, the functions and molecular partners of many maize CMLs remain unknown. Our previous genome-wide analysis of the maize CaM/CML family identified *ZmCML36* among several drought-responsive genes through RNA-seq and qRT-PCR [28]. We selected *ZmCML36* for functional characterization to test whether this stress-responsive expression was associated with a role in stress tolerance. In this study, we evaluated the function of *ZmCML36* in *Arabidopsis* and maize under drought-related osmotic stress and salt stress and examined the Ca^2+^-binding properties of the encoded protein in vitro. We also screened for ZmCML36-interacting proteins and validated its association with Zm00001eb395720, a putative regulatory β subunit of the maize SnRK1 complex. These results provide insight into the role of *ZmCML36* in maize abiotic stress tolerance and suggest a possible connection between Ca^2+^ signaling and SnRK1-associated regulation.

## 2. Results

### 2.1. Identification, Bioinformatic Analysis, and Stress-Induced Expression of ZmCML36

**Figure 1:**
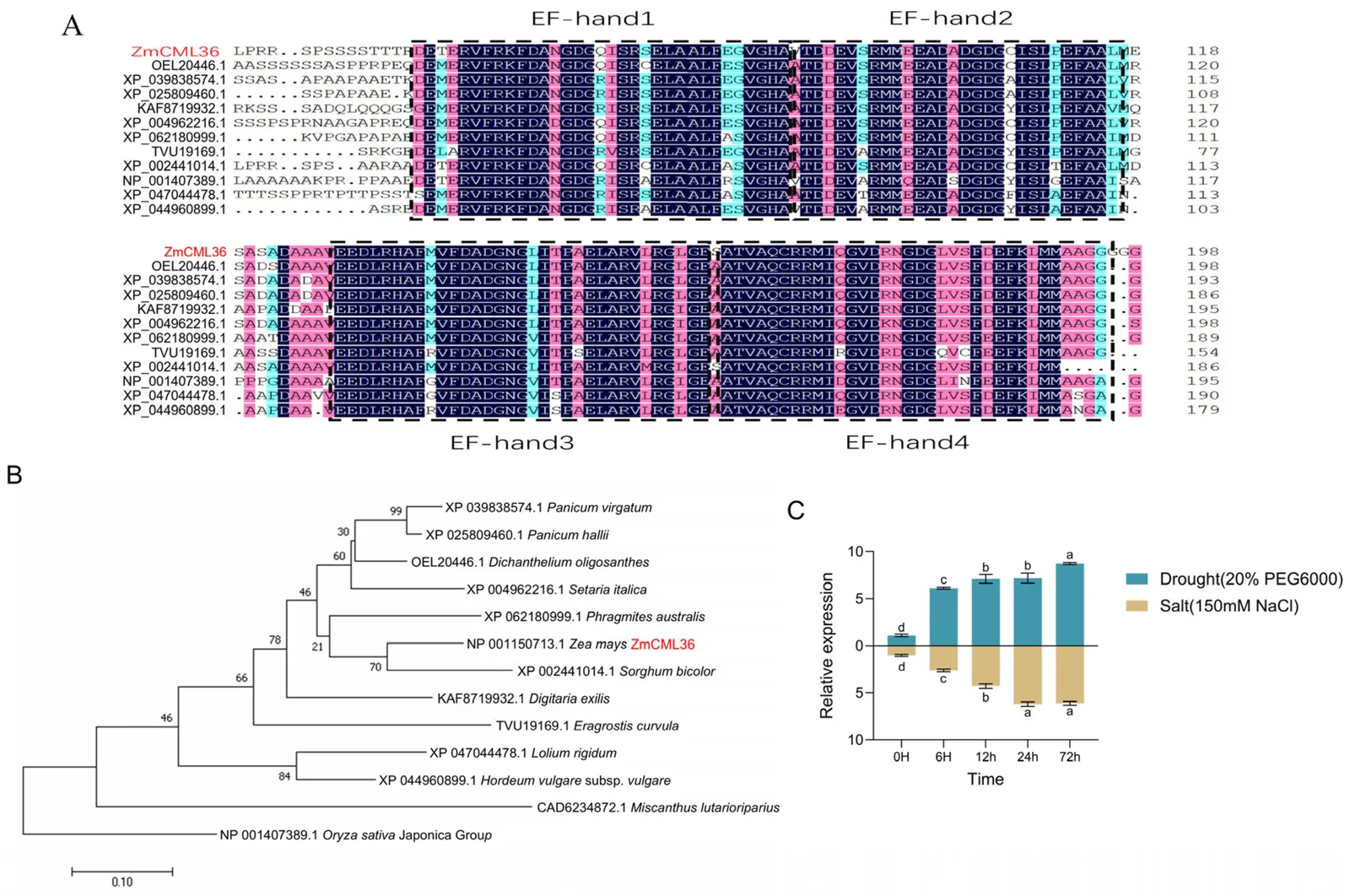
Bioinformatic analysis and stress-responsive expression of *ZmCML36*. (**A**) Multiple sequence alignment of ZmCML36 and homologous proteins from other plant species. ZmCML36 is shown in red. Colors indicate amino acid conservation: black, 100% identity; pink, >75% similarity; and blue, 50–75% similarity. (**B**) Neighbor-joining phylogenetic tree of ZmCML36 and CML proteins from 12 other plant species, constructed using MEGA7. ZmCML36 is shown in red. (**C**) Relative *ZmCML36* transcript abundance in B73 maize treated with 20% PEG6000 or 150 mM NaCl for 0, 6, 12, 24, and 72 h. *ZmTUB* was used as the reference gene, and the 0 h value was used for normalization. Data are presented as the mean ± SD of at least three independent biological replicates. Different letters indicate significant differences according to one-way ANOVA (*p* < 0.05). Homolog protein accessions are shown beside the alignment rows; the study sequence is labeled ZmCML36. The alignment includes 11 homologs, whereas the tree includes 12 homologs.

*ZmCML36* (gene ID Zm00001eb286870; primer sequences in Table S1) encodes a 204-amino-acid protein containing four conserved EF-hand domains (Figure 1A), consistent with its classification as a typical CML protein. Phylogenetic analysis showed that ZmCML36 clustered most closely with its homolog from sorghum (*Sorghum bicolor*) (Figure 1B). Analysis of the 2000-bp region upstream of *ZmCML36* using PlantCARE identified several putative cis-regulatory elements associated with hormone responses and abiotic stress (Table S2), suggesting that *ZmCML36* transcription may respond to environmental signals.

The expression pattern of *ZmCML36* was examined by qRT-PCR in B73 maize seedlings exposed to osmotic and salt stress. Under 20% PEG6000 treatment, *ZmCML36* transcript abundance increased over time and reached its highest level at 72 h (Figure 1C). Under 150 mM NaCl treatment, *ZmCML36* transcript abundance increased markedly at 24 h and remained elevated at 72 h (Figure 1C). These results indicate that *ZmCML36* responds transcriptionally to PEG6000-induced osmotic stress and salt stress.

### 2.2. ZmCML36 Overexpression Enhances Osmotic and Salt Stress Tolerance in Arabidopsis

To evaluate the function of *ZmCML36*, the gene was first expressed heterologously in *Arabidopsis*. Two independent *ZmCML36*-overexpressing Arabidopsis lines, OE6 and OE12, were used for subsequent phenotypic analyses.

**Figure 2:**
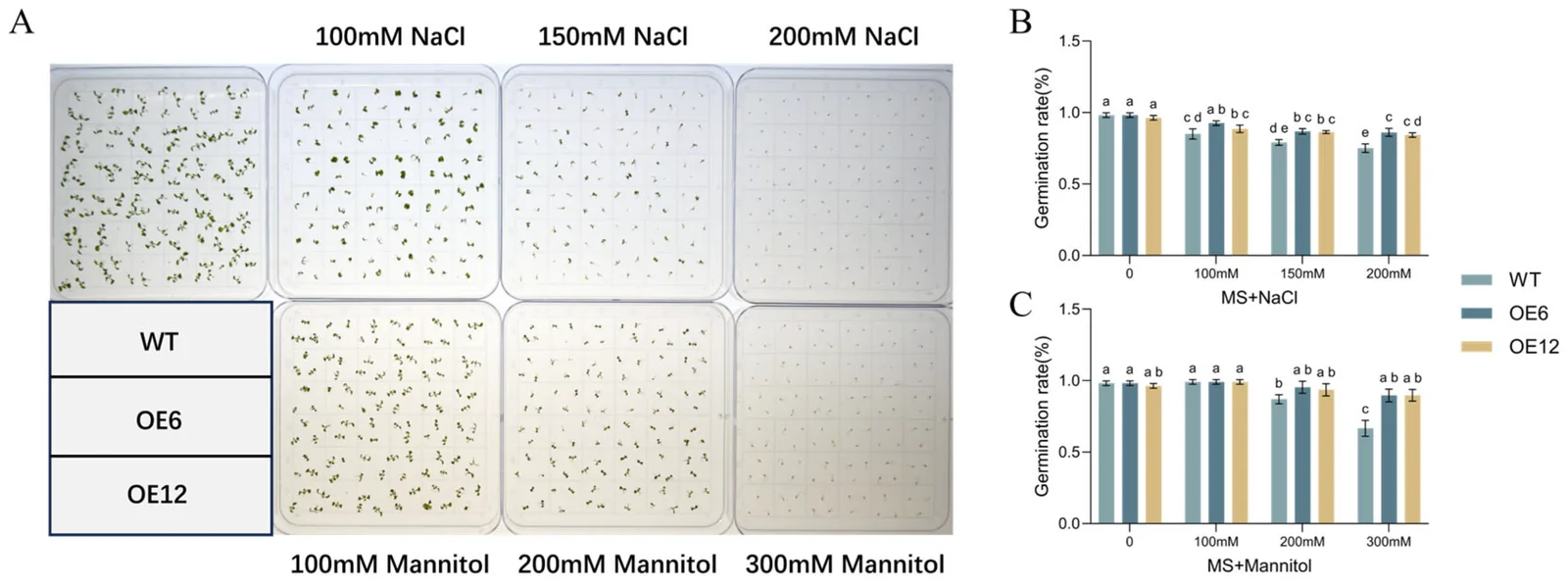
*ZmCML36* overexpression enhances *Arabidopsis* seed germination under salt and mannitol-induced osmotic stress. (**A**) Germination of wild-type (WT), OE6, and OE12 seeds after 10 days on MS medium containing the indicated concentrations of NaCl or mannitol. (**B**,**C**) Germination rates under NaCl and mannitol treatments, respectively. Data are presented as the mean ± SD of at least three independent biological replicates. Different letters indicate significant differences according to two-way ANOVA (*p* < 0.05).

**Figure 3:**
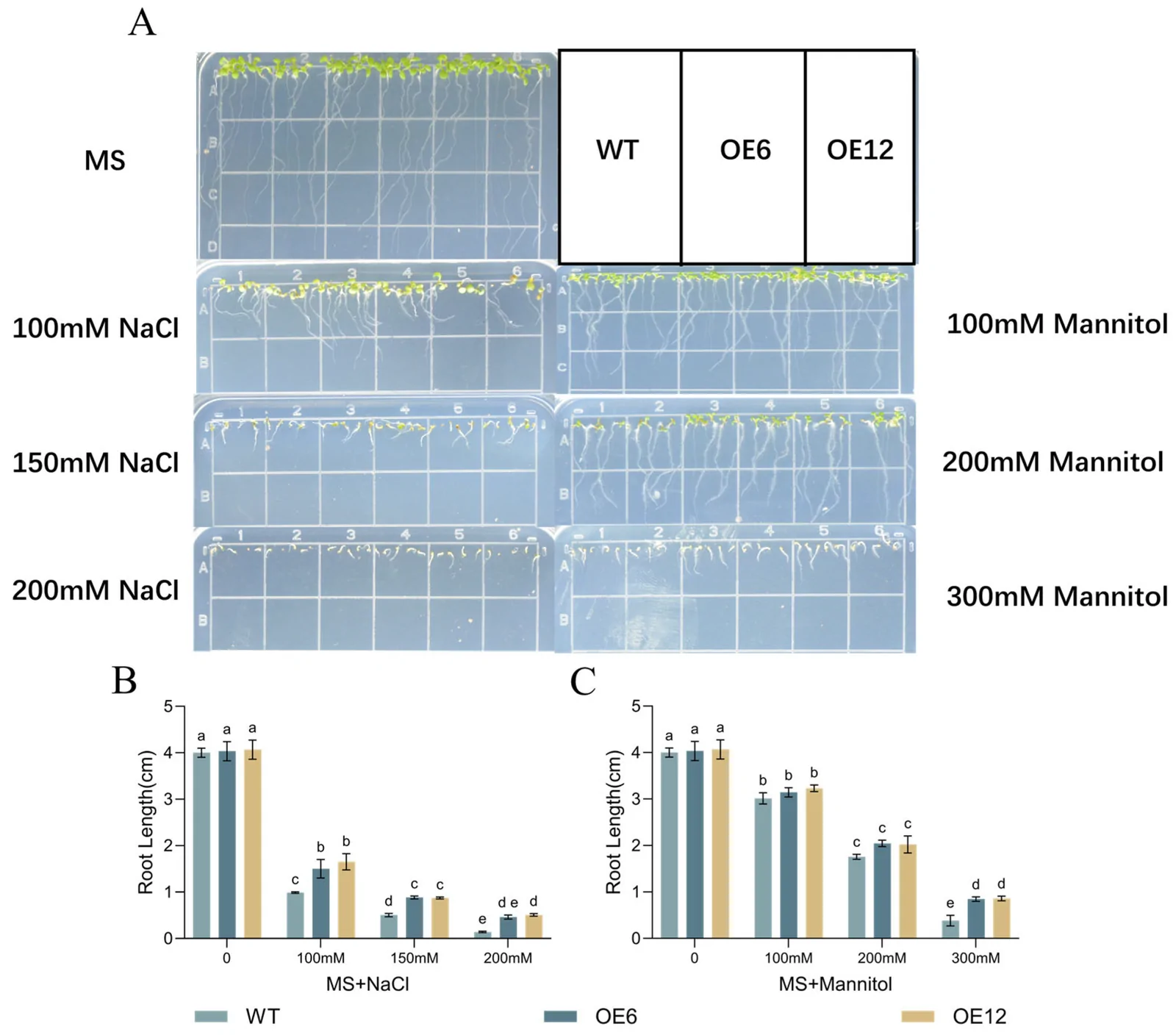
*ZmCML36* overexpression promotes root growth in *Arabidopsis* under salt and mannitol-induced osmotic stress. (**A**) Root growth of WT, OE6, and OE12 seedlings after 10 days on MS medium containing the indicated concentrations of NaCl or mannitol. (**B**,**C**) Root lengths under NaCl and mannitol treatments, respectively. Data are presented as the mean ± SD of at least three independent biological replicates. Different letters indicate significant differences according to two-way ANOVA (*p* < 0.05).

The stress tolerance of transgenic *Arabidopsis* was evaluated during germination and early seedling growth. On MS medium containing different concentrations of NaCl or mannitol, OE6 and OE12 had higher germination rates than wild-type plants. Increasing NaCl or mannitol concentrations progressively inhibited wild-type germination, whereas the overexpression lines retained greater germination capacity (Figure 2A–C). Root growth was also less inhibited in the overexpression lines than in the wild type under both treatments (Figure 3A–C). These results indicate that *ZmCML36* overexpression enhances tolerance to salt and mannitol-induced osmotic stress during early *Arabidopsis* development.

### 2.3. ZmCML36 Positively Regulates Osmotic and Salt Stress Tolerance in Maize

To evaluate *ZmCML36* function in maize, overexpression and EMS-derived mutant materials were analyzed. Stable *ZmCML36*-overexpressing maize lines were generated in the H99 background. Three lines, OE1, OE2, and OE3, were identified by Bar immunostrips and qRT-PCR (Figure S1A,B). An EMS-derived *ZmCML36* mutant in the B73 background was obtained from the maizeEMSDB collection. Sequence analysis identified a GGC-to-GTC codon substitution resulting in a Gly-to-Val change at residue 104 (G104V), and qRT-PCR showed lower *ZmCML36* transcript abundance in the mutant than in wild-type B73 (Figure S1C,D).

**Figure 4:**
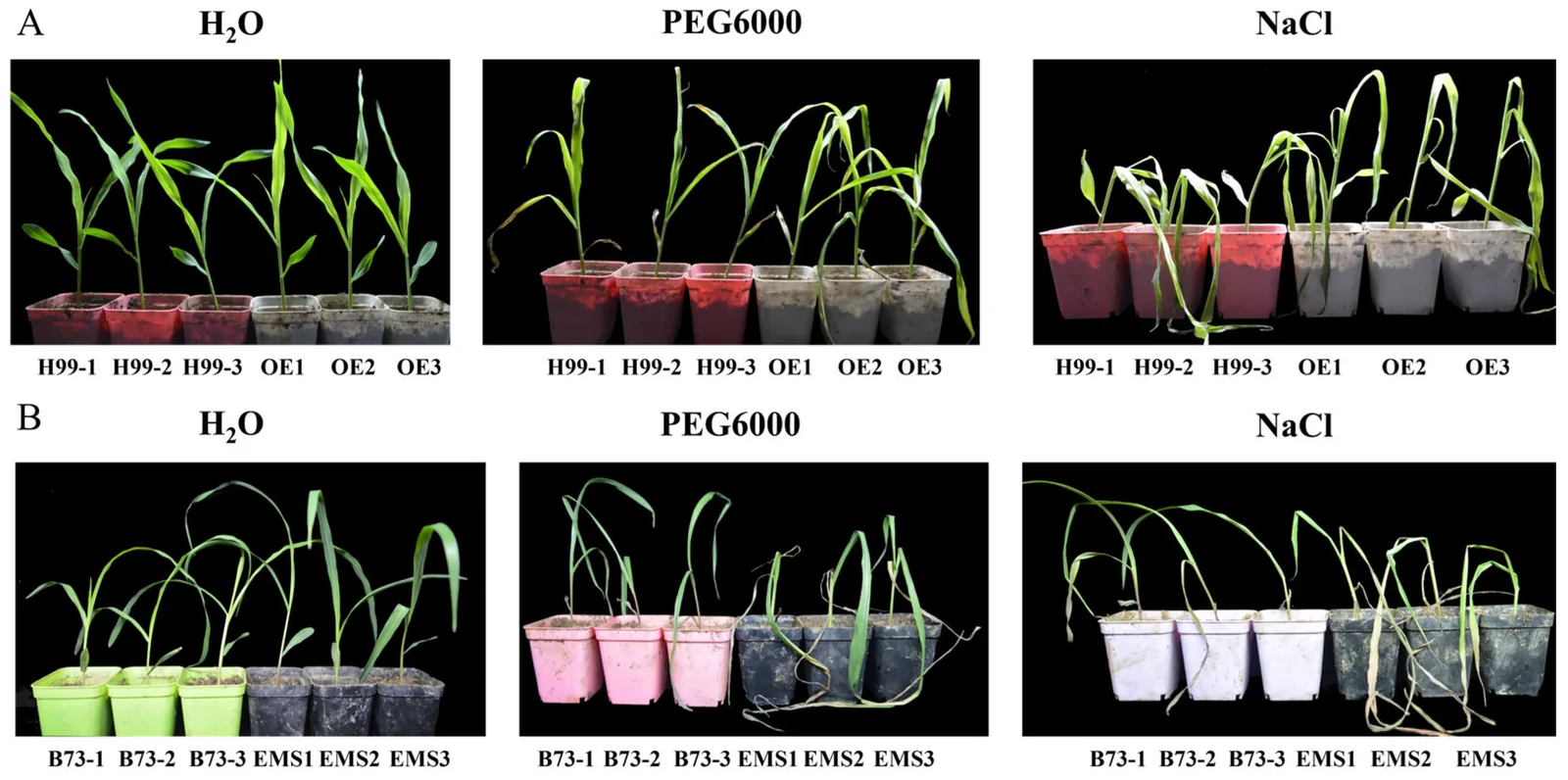
*ZmCML36* contributes to osmotic and salt stress tolerance in maize. (**A**) Phenotypes of three independent *ZmCML36*-overexpressing maize lines (OE1, OE2, and OE3) and their H99 wild-type controls (H99-1, H99-2, and H99-3). (**B**) Phenotypes of *ZmCML36* EMS mutant plants (EMS1, EMS2, and EMS3) and their B73 wild-type controls (B73-1, B73-2, and B73-3). All plants were subjected to H_2_O, 20% PEG6000, or 150 mM NaCl treatments for 10 days.

The maize materials were subjected to stress treatments at the three-leaf stage. After 10 days of treatment with 20% PEG6000 or 150 mM NaCl, all three independent *ZmCML36*-overexpressing maize lines (OE1, OE2, and OE3) exhibited less leaf wilting and yellowing and maintained better overall growth than the H99 wild-type control (Figure 4A). In contrast, the 36-EMS mutant showed more severe wilting, yellowing, and plant death than its B73 wild-type control (Figure 4B). These contrasting phenotypes support a positive role for *ZmCML36* in maize tolerance to osmotic and salt stress.

**Figure 5:**
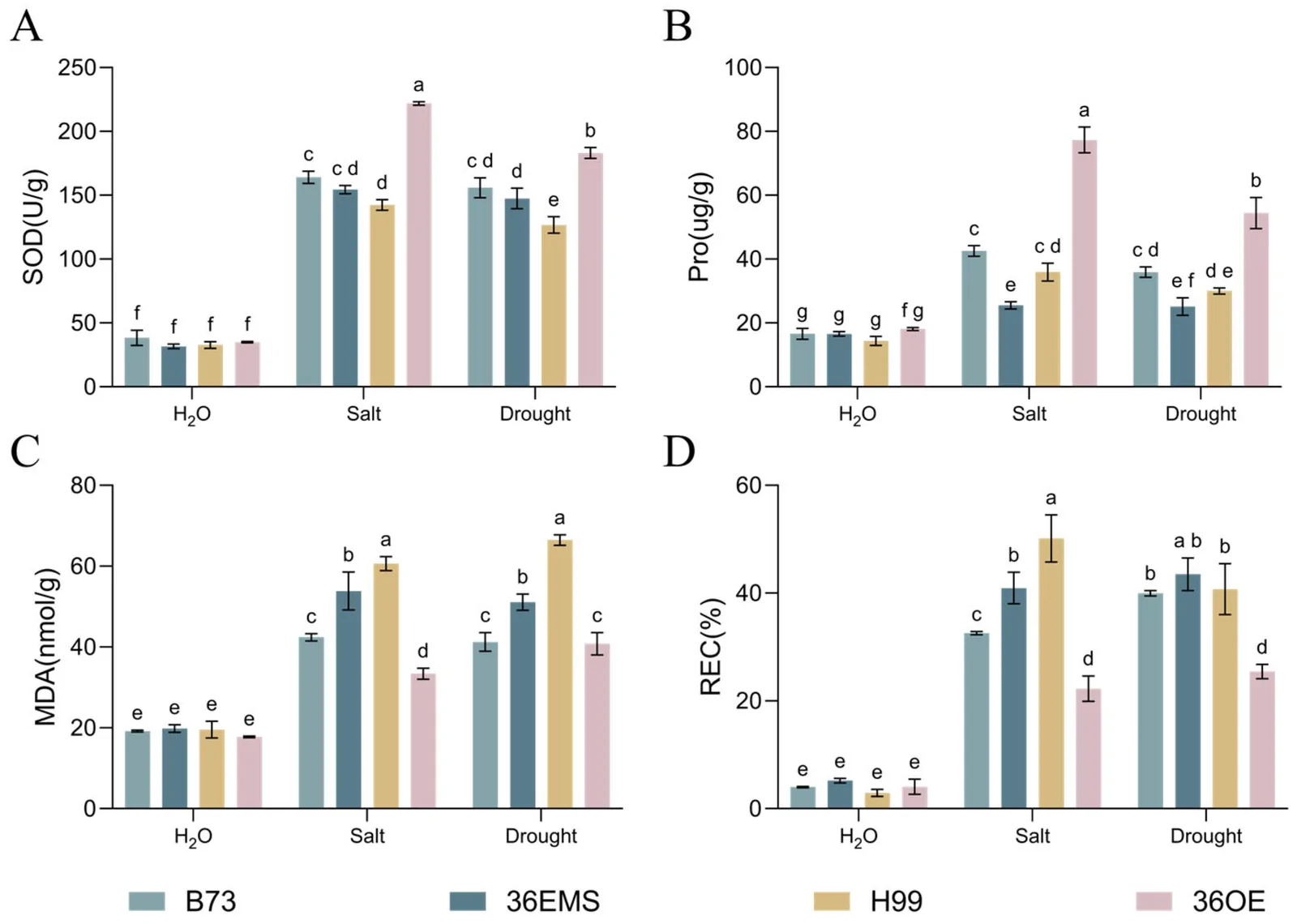
Physiological indices of four maize materials after 10 days of stress treatment. (**A**) SOD activity. (**B**) Proline content. (**C**) MDA content. (**D**) Relative electrical conductivity. For each measurement, 0.5 g of tissue was collected from the second leaf of B73, 36-EMS, H99, and 36-OE plants treated with water, 20% PEG6000, or 150 mM NaCl. Measurements were made separately in OE1, OE2, and OE3 and summarized as 36-OE. The 36-OE materials and 36-EMS mutant were compared with their corresponding H99 and B73 wild-type controls, respectively. Data are presented as the mean ± SD of three independent biological replicates (n = 3), each calculated from the average of three technical measurements. Different letters indicate significant differences according to two-way ANOVA followed by Tukey’s multiple comparisons test (*p* < 0.05).

Physiological and biochemical indices associated with stress responses were then measured. Under PEG6000 and NaCl treatments, the measurements from OE1, OE2, and OE3, summarized as 36-OE, showed higher SOD activity and proline content but lower MDA content and REC than the H99 control. The 36-EMS mutant showed the opposite trends relative to its B73 control (Figure 5A–D).

### 2.4. ZmCML36 Binds Ca^2+^ In Vitro and Shows Fluorescence at the Cell Periphery and Nuclear Region

**Figure 6:**
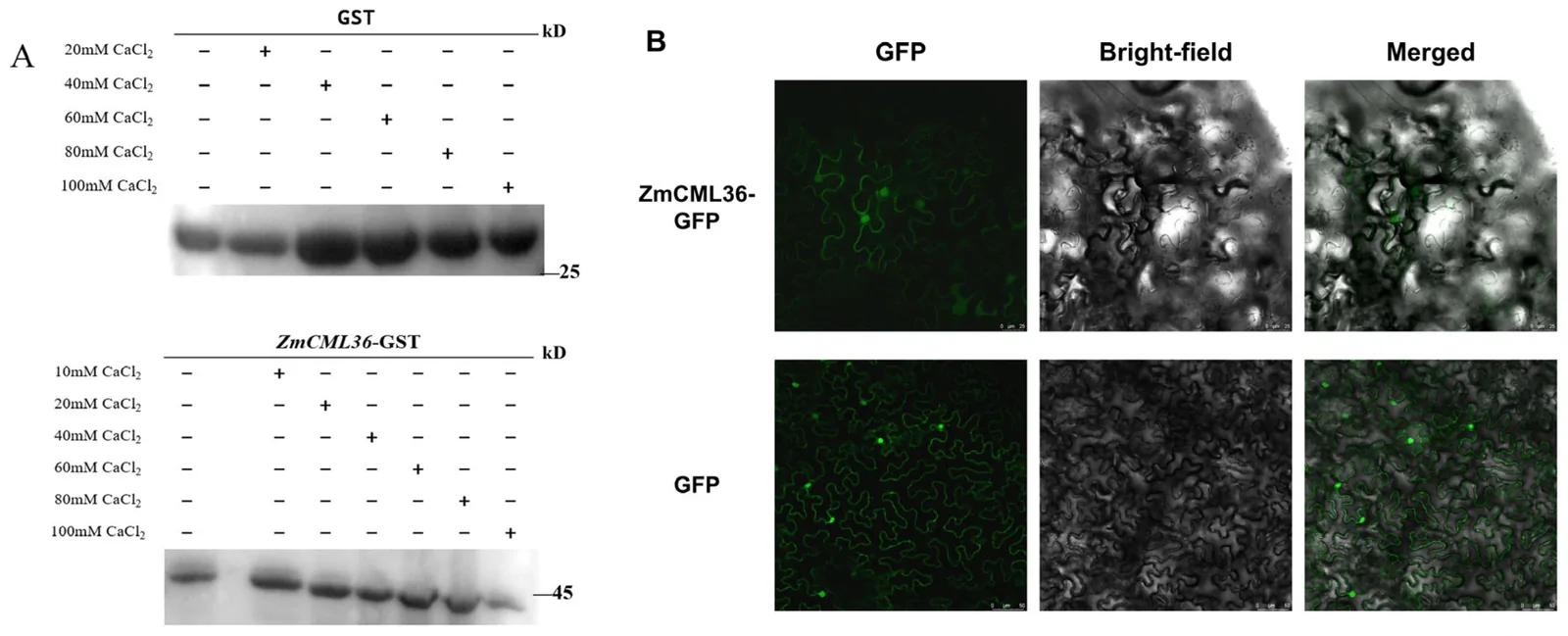
Ca^2+^ binding and subcellular distribution of ZmCML36. (**A**) SDS-PAGE mobility-shift analysis of ZmCML36-GST and GST in the presence of different CaCl_2_ concentrations (10–100 mM for ZmCML36-GST). Proteins were separated by 12% SDS-PAGE and stained with Coomassie Brilliant Blue. Plus and minus signs indicate the presence and absence of the specified component, respectively. Molecular masses are shown in kDa. (**B**) Confocal images of ZmCML36-GFP and the GFP-alone control in *N. benthamiana* epidermal cells (GFP, Bright-field and Merged). Both GFP-channel images received identical global brightness and contrast adjustments for display. Scale bars: 25 μm for ZmCML36-GFP and 50 μm for the GFP control.

To examine the biochemical function of ZmCML36 as a Ca^2+^ sensor, a recombinant ZmCML36-GST fusion protein was expressed and purified from *E. coli*. In an SDS-PAGE mobility-shift assay, the migration of ZmCML36-GST changed as the Ca^2+^ concentration increased, whereas GST migration was unaffected (Figure 6A). These results support the ability of ZmCML36 to bind Ca^2+^ in vitro.

To examine the subcellular distribution of ZmCML36, a ZmCML36-GFP fusion protein was transiently expressed in *N. benthamiana* leaves. Confocal microscopy showed ZmCML36-GFP fluorescence at the cell periphery and in the nuclear region (Figure 6B).

### 2.5. ZmCML36 Interacts with a Putative Regulatory β Subunit of the Maize SnRK1 Complex

To identify potential ZmCML36-interacting proteins, ZmCML36 was used as bait to screen a cDNA library previously constructed in our laboratory from drought-treated B73 maize. After multiple rounds of screening and sequence analysis, 22 candidate interacting proteins were identified (Table S3). Zm00001eb395720 was selected for further analysis and was annotated as a putative regulatory β subunit of the maize SnRK1 complex.

**Figure 7:**
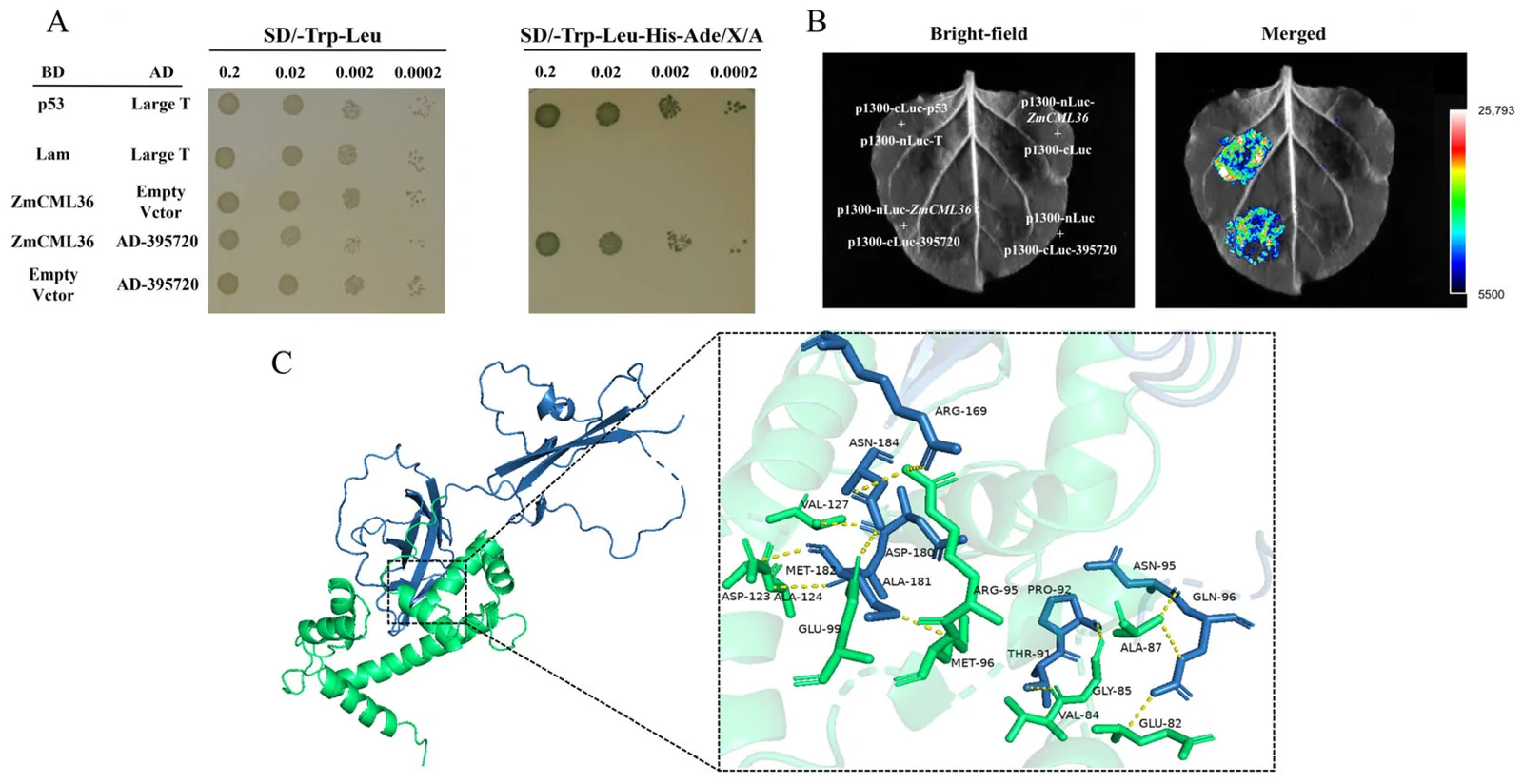
ZmCML36 interacts with Zm00001eb395720, a putative regulatory β subunit of the maize SnRK1 complex. (**A**) Pairwise yeast two-hybrid assay. Yeast cells containing the indicated plasmids were grown on transformation-selection medium (SD/−Trp/−Leu) or interaction-selection medium (SD/−Trp/−Leu/−His/−Ade). The inoculated dilution series is shown above the plates. p53 and T served as the positive control; the remaining combinations, other than ZmCML36 and Zm00001eb395720, served as negative controls. (**B**) Luciferase complementation imaging assay showing the association of ZmCML36 with Zm00001eb395720 in *N. benthamiana* leaves. Brighter luminescence indicates a stronger complementation signal. (**C**) Predicted docking model of ZmCML36 (green) and Zm00001eb395720 (blue). The inset shows the predicted interface, and yellow lines indicate potential hydrogen bonds.

The interaction between ZmCML36 and Zm00001eb395720 was first tested in a pairwise yeast two-hybrid assay. Yeast cells cotransformed with pGBKT7-ZmCML36 and pGADT7-Zm00001eb395720 grew and turned blue on high-stringency selective medium, whereas the negative controls did not grow (Figure 7A). These results support an interaction between the two proteins in yeast.

The interaction was further examined in plant cells using a luciferase complementation imaging assay. Coexpression of nLUC-ZmCML36 and cLUC-Zm00001eb395720 in *N. benthamiana* leaves produced a strong luminescence signal, whereas the negative-control combinations did not (Figure 7B). Thus, the two proteins also associate in plant cells. AlphaFold2 models of ZmCML36 and Zm00001eb395720 were subsequently used for protein–protein docking with HDOCK. The predicted model suggested a potential interaction interface involving hydrogen bonds and hydrophobic residues (Figure 7C); however, this structural model remains hypothetical.

## 3. Discussion

Calcium signaling plays important roles in plant growth, development, and stress responses [29]. Most Ca^2+^ sensors contain EF-hand domains, and CML proteins constitute a major family of plant calcium sensors [30]. Nevertheless, relatively little is known about the functions of individual CML genes in maize. In this study, *ZmCML36* was selected on the basis of our previous maize CaM/CML family analysis and its drought-responsive expression [28]. Phenotypic analyses of overexpression and EMS-derived mutant materials support a positive role for *ZmCML36* in maize tolerance to PEG6000-induced osmotic stress and salt stress.

Physiological measurements provided further support for this role. Under stress treatments, maize overexpressing *ZmCML36* had higher SOD activity and proline content than the H99 wild-type control (Figure 5A,B). Increased SOD activity is associated with ROS scavenging and reduced oxidative damage [31,32], whereas proline accumulation can contribute to osmotic adjustment [33]. MDA content is an indicator of membrane lipid peroxidation, whereas REC reflects electrolyte leakage and membrane integrity [34]. Their lower values in the overexpression materials relative to H99 are consistent with reduced membrane injury under stress (Figure 5C,D). Conversely, the EMS-derived mutant had higher MDA content and REC than its B73 wild-type control (Figure 5C,D) [35,36,37]. Together, these physiological responses are consistent with enhanced antioxidant capacity and osmotic adjustment in *ZmCML36*-overexpressing maize under stress.

CML family members can function as either positive or negative regulators of plant stress responses. For example, *OsCML4* in rice and *MpCML40* in *Millettia pinnata* have been associated with enhanced salt tolerance, whereas *MtCML40* in *Medicago truncatula* has been reported to negatively regulate salt tolerance [38,39,40]. These contrasting roles suggest functional divergence among CML family members. Characterization of *ZmCML36* therefore expands current understanding of CML functions in maize and identifies a candidate gene for improving abiotic stress tolerance.

The physical association of ZmCML36 with Zm00001eb395720, a putative SnRK1 β regulatory subunit, suggests a potential connection between Ca^2+^ sensing and SnRK1-related regulation. SnRK1 integrates metabolic and stress signals and contributes to the balance between growth and defense. Previous studies have implicated the SnRK1 complex in nutrient signaling, energy metabolism, plant development, disease resistance, drought and cold responses, and abscisic acid signaling [41,42,43,44,45,46,47]. In *Arabidopsis*, β subunits regulate the subcellular distribution of SnRK1α and its metabolic stress responses [43,48]. Association with a regulatory subunit therefore provides a plausible point at which a Ca^2+^-binding protein could connect with energy-related stress regulation.

**Figure 8:**
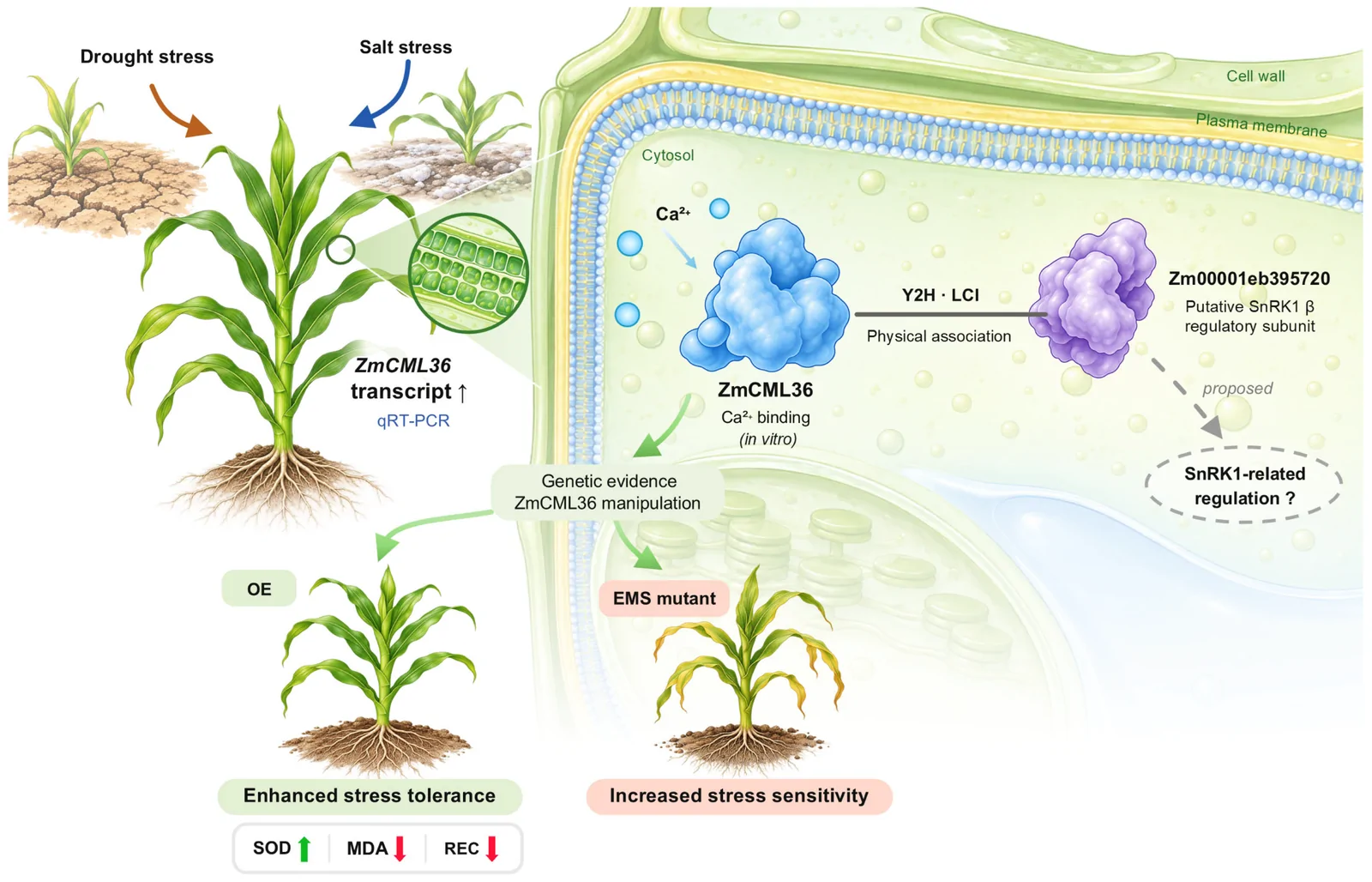
A working model for the role of *ZmCML36* in stress responses. Osmotic stress induced by PEG6000 and salt stress induced by NaCl increased ZmCML36 transcript abundance in maize. *ZmCML36* exhibited Ca^2+^-binding activity in vitro and physically associated with *Zm00001eb395720*, annotated as a putative SnRK1 β regulatory subunit, as supported by yeast two-hybrid (Y2H) and luciferase complementation imaging (LCI) assays. *ZmCML36*-overexpressing maize showed enhanced stress tolerance, whereas the EMS-derived mutant showed increased stress sensitivity relative to their respective wild-type controls. Higher superoxide dismutase (SOD) activity, lower malondialdehyde (MDA) content, and lower relative electrical conductivity (REC) in the overexpression materials are shown as parallel physiological responses. The solid, non-directional line denotes experimentally supported physical association, whereas the gray dashed branch indicates a proposed connection to SnRK1-related regulation that remains to be functionally validated. Colored solid arrows summarize the illustrated stress responses, and upward and downward arrows indicate increases and decreases, respectively.

The expression, Ca^2+^-binding, protein-association, and genetic evidence support a working model for the role of ZmCML36 in stress responses (Figure 8). *ZmCML36* transcript accumulation under PEG6000 and NaCl treatments and the contrasting overexpression and EMS-derived mutant phenotypes support its positive contribution to stress tolerance. The association with Zm00001eb395720 motivates a proposed SnRK1-related branch, while Y2H and LCI establish physical association and docking suggests a possible interface. These findings do not establish SnRK1 activation or Ca^2+^-dependent enhancement of the association.

Functional analysis of the ZmCML36–Zm00001eb395720 association will clarify whether it influences SnRK1 activity and contributes to the observed stress phenotypes.

## 4. Materials and Methods

### 4.1. Plant Materials and Growth Conditions

The plant materials used in this study included the maize inbred lines B73 and H99, wild-type *Arabidopsis thaliana* (Col-0), and *Nicotiana benthamiana*. All materials were provided by the Maize Breeding Innovation Team of Jilin Agricultural University. The maize *ZmCML36* EMS mutant was obtained from the Maize EMS Mutant Library [49].

Seeds of *A. thaliana* and *N. benthamiana* were surface-sterilized with 75% ethanol for 1 min, followed by 5% sodium hypochlorite for 5 min, and then rinsed five times with sterile distilled water. The sterilized seeds were sown on Murashige and Skoog (MS) medium containing 0.8% (*w*/*v*) agar. After stratification in darkness at 4 °C for 3 days, the plates were transferred to a growth chamber under a 16 h light/8 h dark photoperiod at 23 °C/22 °C and a light intensity of 120 μmol m^−2^ s^−1^. Two-week-old seedlings were transplanted into a 3:1 (*v*/*v*) mixture of nutrient soil and vermiculite and maintained under the same conditions.

Maize seeds were surface-sterilized as described above, germinated on moist filter paper at 28 °C for 48 h, and transplanted into the same soil mixture. Maize plants were grown in a growth chamber under a 16 h light/8 h dark photoperiod at 28 °C/25 °C.

### 4.2. RNA Extraction and Quantitative Real-Time PCR Analysis

Total RNA was extracted using TRIGene Reagent (Kangrun Biotech, Beijing, China). First-strand cDNA was synthesized using the ReverTra Ace^®^ qPCR RT Kit (TOYOBO, Osaka, Japan). Quantitative real-time PCR (qRT-PCR) was performed using 2× RealStar Fast SYBR qPCR Mix on a QuantStudio™ 3 system (Thermo Fisher Scientific, Waltham, MA, USA). *ZmTUB* (Zm00001eb218000) and *AtACTIN2* (AT3G18780) were used as reference genes for maize and *Arabidopsis*, respectively. Relative transcript abundance was calculated using the 2^−ΔΔCt^ method from three biological replicates, each measured in three technical replicates.

### 4.3. Cloning and Bioinformatic Analysis of ZmCML36

The coding sequence (CDS) of *ZmCML36* was amplified from B73 maize cDNA using the primers listed in Table S1. Homologous protein sequences were aligned using DNAMAN version 9. A phylogenetic tree was constructed using the neighbor-joining method in MEGA7. Cis-regulatory elements in the *ZmCML36* promoter were predicted using PlantCARE (https://bio.tools/plantcare (accessed on 24 March 2024)).

### 4.4. Analysis of ZmCML36 Expression Under Abiotic Stress

Maize seedlings at the three-leaf stage were treated with 150 mM NaCl or 20% PEG6000. Leaf samples were collected at 0, 6, 12, 24, 48, and 72 h for qRT-PCR analysis. The primers are listed in Table S1.

### 4.5. Generation and Identification of Transgenic Plants

The *ZmCML36* CDS was cloned into pCAMBIA3301 using the NcoI and PmlI sites for overexpression in *Arabidopsis* and into a modified pCAMBIA3301 vector containing the maize ubiquitin promoter using the BamHI and MluI sites for overexpression in maize. The primers are listed in Table S1. The constructs were introduced into *Agrobacterium tumefaciens* GV3101 for *Arabidopsis* transformation and EHA105 for maize transformation. *Arabidopsis* was transformed using the floral-dip method [50], and maize inbred line H99 was transformed using an *Agrobacterium*-mediated method [51]. Arabidopsis transformants were selected using 0.1% glufosinate after floral-dip transformation and propagated to the T3 generation. Two independent overexpression lines, OE6 and OE12, were used for subsequent assays. Maize plants carrying pCAMBIA3301-ubi-ZmCML36 were selected using 0.1% glufosinate at the three-leaf stage in the T0 and T1 generations. T2 plants were verified using Bar immunostrips and qRT-PCR, and three overexpression lines, OE1, OE2, and OE3, were retained for subsequent assays (Figure S1A,B).

### 4.6. Stress-Tolerance Assays and Measurement of Physiological Indices

Transgenic *Arabidopsis* seeds were germinated on MS medium containing different concentrations of NaCl or mannitol. Germination rates and root lengths were recorded after 10 days. For soil-based assays, *Arabidopsis* and maize seedlings were treated with 150 mM NaCl or 20% PEG6000 for 10 days. Maize seedlings were treated at the three-leaf stage at 28 °C. Treatment solutions were applied by irrigation until the potting soil was thoroughly moistened and reapplied approximately every 3 days; water-treated plants served as controls. The stated concentrations refer to the applied treatment solutions; PEG6000 was used to impose osmotic stress. Physiological measurements were made separately in OE1, OE2, and OE3 and summarized collectively as 36-OE. The 36-OE materials were compared with their H99 wild-type control, whereas the 36-EMS mutant was compared with B73. Relative electrical conductivity (REC), malondialdehyde (MDA) content, proline content, and superoxide dismutase (SOD) activity were measured using commercial assay kits (Solarbio, Beijing, China).

### 4.7. Subcellular Localization

The *ZmCML36* CDS was fused in frame with mGFP5 in the pCAMBIA1302 vector using the NcoI and BglII sites; the primers are listed in Table S1. The construct was transiently expressed in *N. benthamiana* leaves by *Agrobacterium*-mediated infiltration with strain GV3101. GFP fluorescence was observed using a laser-scanning confocal microscope (Leica, Frankfurt, Germany). GFP alone expressed from the empty pCAMBIA1302 vector served as the control.

### 4.8. Recombinant Protein Expression and In Vitro Calcium-Binding Assay

The *ZmCML36* CDS was cloned into pGEX-4T-1 using the BamHI and XhoI sites for recombinant expression; the primers are listed in Table S1. The GST-tagged protein was expressed in *Escherichia coli* BL21 (DE3) and purified. For the Ca^2+^-binding assay, approximately 10 μg of purified protein was incubated at 30 °C for 1 h in a final reaction volume of 20 μL containing 50 mM Tris-HCl (pH 7.5) and CaCl_2_ at final concentrations of 10–100 mM. Ca^2+^-dependent mobility shifts were analyzed by 12% SDS-PAGE.

### 4.9. Yeast Two-Hybrid Assay

To screen for proteins that interact with ZmCML36, the *ZmCML36* CDS was cloned into the NdeI and BamHI sites of pGBKT7 to generate the bait construct pGBKT7-ZmCML36; the primers are listed in Table S1. The bait plasmid was transformed into the Y2HGold strain, and the absence of autoactivation and toxicity was confirmed on SD/−Trp medium containing X-α-Gal and aureobasidin A. A cDNA library previously constructed in our laboratory from drought-treated B73 maize was used for screening. Positive clones were selected on SD/−Leu/−Trp/−His/−Ade medium containing X-α-Gal and aureobasidin A. Prey plasmids were recovered from positive clones and sequenced. For pairwise validation, the bait and prey plasmids were cotransformed into Y2HGold, and yeast growth was evaluated on selective media of increasing stringency.

### 4.10. Luciferase Complementation Imaging Assay

To validate the protein interaction in plant cells, the CDSs of *ZmCML36* and *Zm00001eb395720* were cloned into the pCAMBIA1300-nLUC and pCAMBIA1300-cLUC vectors, respectively, using the BamHI and MluI sites; the primers are listed in Table S1. *Agrobacterium tumefaciens* GV3101 strains carrying the nLUC and cLUC fusion constructs were coinfiltrated into *N. benthamiana* leaves. After 3 days, 1 mM D-luciferin potassium salt was infiltrated into the same leaf areas. Following incubation in darkness for 5 min, luminescence was detected using a Tanon-5200 chemiluminescence imaging system (Tanon, Shanghai, China).

### 4.11. Protein Structure Modeling and Docking

Three-dimensional models of ZmCML36 and Zm00001eb395720 were predicted using AlphaFold2. Protein–protein docking was performed using the HDOCK server (http://hdock.phys.hust.edu.cn/ (accessed on 24 March 2024)), and the resulting models were visualized and analyzed using PyMOL version 2.5.

### 4.12. Statistical Analysis

All experiments included at least three independent biological replicates. Data are presented as the mean ± standard deviation (SD). Statistical analyses were performed using GraphPad Prism 9. For the physiological measurements shown in Figure 5, three independent biological replicates were analyzed per group, with each biological replicate represented by the mean of three technical measurements. These data were analyzed using two-way ANOVA followed by Tukey’s multiple comparisons test. The statistical tests used for individual experiments are specified in the corresponding figure legends, and differences were considered significant at *p* < 0.05.

## 5. Conclusions

The maize calmodulin-like protein ZmCML36 positively contributes to drought-related osmotic stress and salt stress tolerance. ZmCML36 binds Ca^2+^ in vitro, and its GFP fusion shows fluorescence at the cell periphery and nuclear region. ZmCML36 also interacts with Zm00001eb395720, a putative regulatory β subunit of the maize SnRK1 complex. This association suggests a potential connection between Ca^2+^ signaling and SnRK1-associated regulation during abiotic stress. *ZmCML36* is therefore a candidate gene for improving abiotic stress tolerance in maize.

## Data Availability

All data generated or analyzed during this study are included in this published article and its Supplementary Materials Files.

## Supplementary Materials

The following supporting information can be downloaded at https://www.mdpi.com/article/10.3390/plants15213409/s1, Figure S1: Identification of transgenic and EMS-derived *ZmCML36* maize materials; Table S1: Primers and sequences used in this study; Table S2: Predicted cis-regulatory elements in the 2000-bp region upstream of *ZmCML36*; Table S3: Candidate *ZmCML36*-interacting proteins identified in a yeast two-hybrid screen.
