# Supplementary material for: The Maize Calcium-Binding Protein ZmCML36 Positively Regulates Tolerance to Drought and Salt Stress
Source: Plants (Basel). 2026 Nov 2;15(21):3409. doi: 10.3390/plants15213409 (PMC13765929; doi:10.3390/plants15213409)
Supplement: Supplementary file 1 [file plants-15-03409-s001.zip › Supplementary Figure S1.pdf]

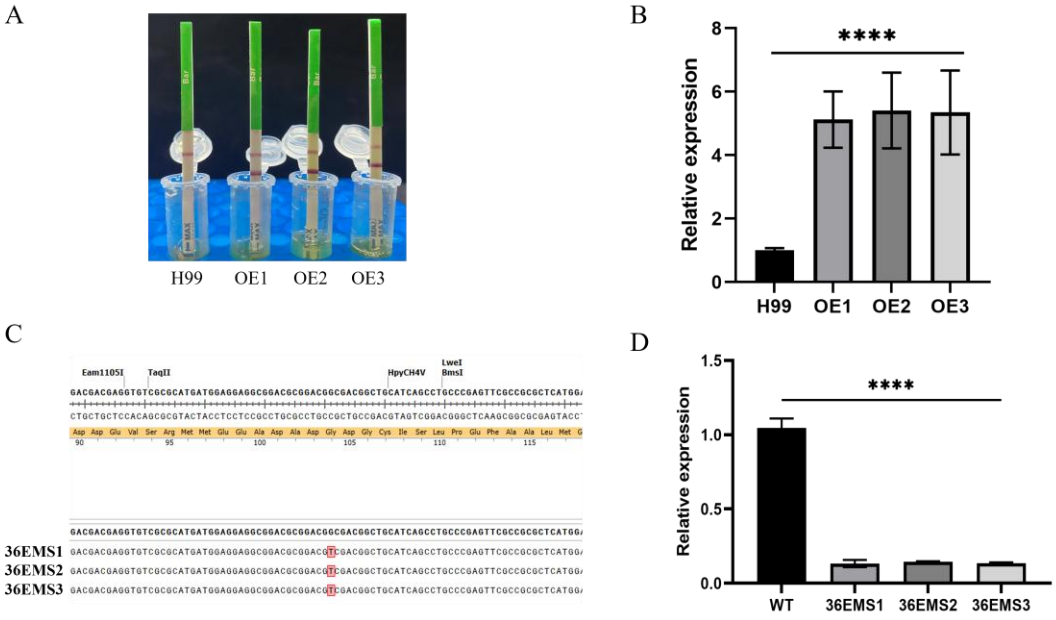

Figure S1. Identification of transgenic and EMS-derived *ZmCML36* maize materials. (A) Bar immunostrip analysis of the T2 *ZmCML36*-overexpressing maize lines OE1, OE2, and OE3 and the H99 wild-type control; two bands indicate a positive transgenic plant. (B) Relative *ZmCML36* transcript abundance in OE1, OE2, OE3, and H99 determined by qRT-PCR, with *ZmTUB* as the reference gene and H99 used for normalization. (C) Sequence identification of the EMS-derived *ZmCML36* mutant. Genomic DNA from the mutant samples and wild-type B73 was amplified by PCR and sequenced using the primers listed in Table S1. The highlighted substitution changes codon GGC to GTC, resulting in a Gly-to-Val substitution at amino acid 104 (G104V). (D) Relative *ZmCML36* transcript abundance in the mutant lines and B73 determined by qRT-PCR, with *ZmTUB* as the reference gene and B73 used for normalization. Data in (B,D) are presented as the mean ± SD of at least three independent biological replicates. Statistical significance was assessed using Student's t-test; **** $p < 0.0001$.
