# Supplementary material for: The Maize Calcium-Binding Protein ZmCML36 Positively Regulates Tolerance to Drought and Salt Stress
Source: Plants (Basel). 2026 Nov 2;15(21):3409. doi: 10.3390/plants15213409 (PMC13765929; doi:10.3390/plants15213409)
Supplement: Supplementary file 1 [file plants-15-03409-s001.zip › Supplementary Tables S1-S3.pdf]

Table S1. Primers and sequences used in this study

Primer name	Forward primer sequence (5′–3′)	Reverse primer sequence (5′–3′)
36-3301	CGGGGGACTCTTGACCATGGCAATGGGCAAGGTTCGGTCC	ACCGTTAATTAACCCCACGTGCGAGGCACCGATCCTGCC
36-3301-ubi	GGATCCATGGATTACAAGGACGACGATGACAAGATGGGCAAGGTTCGG	ACGCGTCTACTAGGCACCGATCCT
36-1302	CGGGGGACTCTTGACCATGGCAATGGGCAAGGTTCGGTCC	TCTCCTTTACTAGTCCGAGATCTGTAGGCACCGATCCT
ZmCML36	ATGGGCAAGGTTCGG	CTACTAGGCACCGATCCT
qPCR	CGAATCTTCAACAGGCAAAGC	ACCGAACCTTGCCCATG
ZmTUB	CTACCTCACGGCATCTGCTATGT	GTCACACACACTCGACTTCACG
AtACTIN2	GCACCACCTGAAAGGAAGTACA	CGATTCCTGGACCTGCCTCATC
36-GST	GATCTGGTTCCGCGTGGATCCATGGGCAAGGTTCGG	GTCACGATGCGGCCGCTCGAGGTAGGCACCGATCCT
36-BD	TCAGAGGAGGACCTGCATATGATGGGCAAGGTTCGG	CCGCTGCAGGTCGACGGATCCCGAGGCACCGATCCT
395720-AD	GTACCAGATTACGCTCATATGATGGGAAACGCCAGCGG	ATGCCCACCCGGGTGGAATTCCTATCGTTCGATGGGTTTGTACAGG
36-nLUC	CGAGCTCGGTACCCGGGATCCATGGGCAAGGTTCGG	AGCCACCGCCCCGGGACGCGTCGTAGGCACCGATCCT
395720-cLUC	GAGGTCAGATCTCGTACGCGTCGATGGGAAACGCCAGCG	TGTAGTCCATTTGTTGGATCCCTATCGTTCGATGGGTTTGT

Table S2. Predicted cis-regulatory elements in the 2,000-bp region upstream of *ZmCML36*

Element	Sequence	Number
AT-rich element	ATAGAAATCAA	1
TC-rich repeats	ATTCTCTAAC	1
LTR	CCGAAA	3
TCA-element	CCATCTTTTT	1
ABRE	ACGTG	6
ARE	AAACCA	3
circadian	CAAAGATATC	1
G-Box	CACGTT	1
	CACGAC	2
	CACGTG	2
O2-site	GATGACATGG	1
CAT-box	GCCACT	3
CAAT-box	CAAAT	20
TATA-box	TATA	4
GARE-motif	TCTGTTG	1
GT1-motif	GGTTAAT	1
MBS	CAACTG	1
CCAAT-box	CAACGG	1
Box 4	ATTAAT	4
GTGGC-motif	CAGCGTGTGGC	1
chs-CMA2a	TCACTTGA	1
GATA-motif	GATAGGG	3

Table S3. Candidate *ZmCML36*-interacting proteins identified in a yeast two-hybrid screen

Gene ID	Description	Length (bp)
Zm00001eb191920	RING-type zinc finger family protein. RING-type zinc finger and von Willebrand factor A domains.	5321
Zm00001eb152680	Putative calcyclin-binding protein. Calcyclin-binding/CS and SGS domains.	5417
Zm00001eb245930	Uncharacterized protein (predicted APC15 family). APC15-family assignment.	770
Zm00001eb273100	Putative BRE1-family E3 ubiquitin-protein ligase. BRE1-family and RING domains.	12159
Zm00001eb065790	Putative phosphomannomutase (EC 5.4.2.8). Predicted mannose-6-phosphate/mannose-1-phosphate interconversion.	3076
Zm00001eb318020	Basic helix–loop–helix (bHLH) domain-containing protein. Predicted DNA-binding/transcriptional-regulatory protein.	2534
Zm00001eb291810	Putative translation initiation factor SUI1 homolog. SUI1-family translation initiation factor.	2459
Zm00001eb269190	DnaJ/Hsp40-family chaperone protein. J domain and DnaJ C-terminal domains.	5245
Zm00001eb102120	Putative endoplasmic reticulum membrane protein complex subunit 3. EMC3/TMCO1-family membrane-protein-biogenesis component.	9660
Zm00001eb205890	DDI1-like ubiquitin-domain-containing protein. Ubiquitin-like and DDI1-type aspartic-peptidase domains.	6529
Zm00001eb228040	Uncharacterized protein (GIL1-like family). GIL1-like family assignment only.	3345
Zm00001eb096920	Putative glycoside hydrolase family 31 protein. GH31 domains and active-site signature.	31510
Zm00001eb245950	Putative NBR1-like protein. NBR1 FW, PB1 and UBA domains.	5188
Zm00001eb140380	Uncharacterized protein; biochemical function unresolved.	2938
Zm00001eb396370	Putative argininosuccinate lyase. Argininosuccinate lyase family.	4338
Zm00001eb250330	Putative serine/threonine-protein kinase (EC 2.7.11.1). Protein kinase domain and serine/threonine active-site signature.	6178
Zm00001eb395720	Putative SnRK1 β regulatory subunit. ASC and carbohydrate-binding-module-related domains.	7025
Zm00001eb089490	Putative NB-ARC/LRR disease-resistance protein. NB-ARC and LRR-related domains.	3631
Zm00001eb328850	Putative ATP-dependent RNA helicase. DEAD/DEAH-box/helicase ATP-binding domains.	8910
Zm00001eb430750	Uncharacterized protein (DUF2358 family). DUF2358 and NTF2-like-fold assignment.	2329
Zm00001eb310130	BURP domain-containing protein. Polygalacturonase-associated BURP family.	2362
Zm00001eb371150	Putative peptidase M28-family protein. M28B peptidase and transferrin-receptor-like dimerization domains.	5172

Candidate proteins were identified in the initial Y2H screen; Zm00001eb395720 was selected for pairwise Y2H and LCI validation. Functional descriptions are putative family/domain annotations based on UniProtKB records with B73 RefGen_v5 transcript cross-references and available InterPro domains (accessed 23 October 2026). Unresolved functions are retained as uncharacterized.
